# Coffee’s Impact on Health and Well-Being

**DOI:** 10.3390/nu17152558

**Published:** 2025-08-05

**Authors:** Ryan C. Emadi, Farin Kamangar

**Affiliations:** 1 School of Medicine, West Virginia University, Morgantown, WV 26506, USA; rce00003@mix.wvu.edu; 2 School of Computer, Mathematical, and Natural Sciences, Morgan State University, Baltimore, MD 21251, USA

**Keywords:** coffee, caffeine, cardiovascular mortality, chronic disease prevention, cancer risk reduction, cognitive decline mitigation, pregnancy and caffeine safety

## Abstract

Coffee is one of the most widely consumed beverages globally, with over 60% of Americans drinking it daily. This review examines coffee’s multifaceted impact on health and well-being, drawing on decades of research. Overall, the consensus is that moderate coffee intake is more beneficial than harmful across a wide range of health outcomes. Numerous large-scale, prospective cohort studies from around the world have consistently shown that moderate coffee consumption—typically three to five cups per day—is associated with reduced overall mortality and lower risk of major diseases such as cardiovascular diseases, diabetes, stroke, respiratory conditions, cognitive decline, and potentially several types of cancer, including liver and uterine cancers. Both caffeinated and decaffeinated coffee have shown benefits. The addition of sugar and cream to coffee may attenuate coffee’s positive health effects. Despite historical concerns, coffee consumption is not linked to increased risks of cancer, hypertension, or arrhythmia. However, some concerns remain. For pregnant women, coffee consumption should be limited to lower amounts, such that the daily intake of caffeine does not exceed 200 mg/day. Also, excessive caffeinated coffee intake may cause anxiety or sleep disturbances. Coffee’s health-promoting mechanisms include improved glucose balancing, increased physical activity, increased fat oxidation, improved lung function, and reduced inflammation. Beyond mortality and chronic diseases, coffee consumption affects many aspects of well-being: it supports hydration, boosts mental acuity, enhances physical performance, and may aid bowel recovery after surgery. While the field is well-studied via long-term observational cohorts, future research should focus on randomized controlled trials, Mendelian randomization studies, and granular analyses of coffee types and additives.

## 1. Introduction

Coffee is one of the most popular drinks in the world, with over 2 billion cups being consumed every day [1]. Consumption is especially high in Western countries, with 66% of Americans drinking coffee every day [2]. The average American coffee drinker consumes three cups per day [2].

Derived from plants native to eastern parts of Africa, coffee made its way to Europe in the 17th century [3]. Brewed coffee gradually became the staple drink of Europeans, replacing beer and wine as a clean source of hydration. The impact of this replacement was perhaps on both taste buds and on mental and physical activity, going from a mild sense of tranquility and inebriation to a sense of sharpened mental acuity and readiness to work. Not surprisingly, coffee became the drink of the Industrial Revolution. It also led to the creation of Penny Universities, where intellectuals gathered and discussed notable issues [4]. In an 1824 letter, Thomas Jefferson called coffee “the favorite beverage of the civilized world” [5]. This fact has not changed for over two centuries after Jefferson’s letter.

Owing to its very high consumption rate and historical significance, the health effects of coffee are very important to clinicians, dietitians, and consumers. Consumers may ask their healthcare providers about the positive and negative effects of coffee on their health, about its mechanisms of action, and about certain circumstances when they should avoid coffee intake. They may also ask about coffee’s impact on certain aspects of well-being, such as sleep or hydration. This paper was written to provide an updated overview of the effects of coffee on *health* and *well-being*. While the focus of this paper is on coffee, we have included some salient studies on the health effects of caffeine intake as well. Coffee consumption is the most important source of caffeine intake [6], and any discussion of the health effects of coffee is inextricably intertwined with those of caffeine.

Several excellent narrative reviews, systematic reviews, and meta-analyses [7,8,9] have previously addressed the health effects of coffee. This review paper provides an updated review of the literature through 2025. Our goal is to provide a comprehensive overview, such that it will be most useful to clinicians, dietitians, and consumers. We will discuss the following:

(a) The most recent ruling by the U.S. Food and Drug Administration (FDA);
(b) The association between coffee consumption and reduced overall mortality;
(c) The effects of coffee on cause-specific mortality and morbidity;
(d) The mechanisms of action of coffee leading to reduced morbidity and mortality;
(e) The health effects of adding sugar and cream to coffee;
(f) Potential areas of concern, such as the effects of coffee on pregnancy or anxiety;
(g) Coffee’s other effects on well-being, such as its effects on hydration or sleep;
(h) Potential future research directions.

## 2. The U.S. Food and Drug Administration’s Final Rule

In its final rule, the U.S. Food and Drug Administration (FDA) recently announced that “coffee with less than 5 calories per Reference Amount Customarily Consumed (RACC)” can automatically qualify for the ‘healthy’ claim [10]. This ruling likely stems from coffee’s relatively low contribution to total calorie intake, as well as its favorable profile across many health outcomes, which have been extensively studied over several decades. These studies form the basis of the present review.

Coffee influences many aspects of health, the gestalt of which is best captured by its impact on overall mortality. Therefore, we emphasize the impact of coffee consumption on reduced overall mortality and correspondingly increased longevity.

## 3. Overall Mortality

The large majority of prospective cohort studies (Table 1) have found that coffee consumption is associated with reduced mortality rates [11,12,13,14,15,16,17,18,19,20,21,22,23]. For example, a pivotal study by Freedman and colleagues, which enrolled over 400,000 individuals from the NIH-AARP Diet and Health Study, showed a 10% to 15% reduction in overall mortality among those who drank two or more cups of coffee per day [15].

These prospective studies, which collectively include several million participants, have been conducted in the United States, Europe, and Asia. Detailed adjustment for confounders and the consistency of results across several nations, with various confounding structures, suggest that the risk reductions are unlikely to be due to confounders. Several of these studies have conducted sensitivity analyses to assess the potential for reverse causality, but the results have remained largely unchanged. For example, Freedman and colleagues found that the associations were equally strong after excluding data from the first 9 years of follow-up and when limiting the results to individuals who reported good to excellent health at baseline, arguing against reverse causality [15].

A 2019 meta-analysis of 40 studies, including 3.8 million individuals, showed non-linear inverse associations between coffee consumption and overall mortality [24]. The lowest relative risk (RR) was at intakes of 3.5 cups/day for all-cause mortality (RR = 0.85, 95% confidence interval: 0.82–0.89). The association between coffee consumption and overall mortality remained stable across strata of age, weight, alcohol drinking, smoking, and caffeine content of coffee.

## 4. Major Causes of Mortality and Morbidity

The most common causes of death in the United States and in most other parts of the world are cardiovascular diseases (including coronary heart disease and stroke), cancer, injuries and accidents, chronic lower respiratory disease, diabetes, chronic liver disease, kidney diseases, cognitive disorders (including Alzheimer’s disease), and Parkinson’s disease [25]. Coffee consumption has shown an inverse association with several of these conditions. For example, Freedman and colleagues studied the association of coffee with cause-specific mortality in the NIH-AARP Cohort and concluded that “inverse associations were observed for deaths due to heart disease, respiratory disease, stroke, injuries and accidents, diabetes, and infections, but not for deaths due to cancer” [15]. Likewise, Loftfield and colleagues reviewed the association of coffee with cause-specific mortality in the PLCO Cohort and concluded that “inverse associations were observed for deaths from heart disease, chronic respiratory diseases, diabetes, pneumonia and influenza, and intentional self-harm, but not cancer” [17].

**Cardiovascular disease:** A meta-analysis of 36 prospective human studies [26], which included nearly 1.3 million participants, found that cardiovascular disease risk was lowest among those who consumed 3–5 cups of coffee per day, corresponding to approximately 300 to 500 mg of caffeine per day, with an almost 15% reduction in risk. Coffee consumption was associated with reduced risk of both coronary heart disease and stroke.

**Cancer**: In 2016, the World Health Organization’s International Agency for Research on Cancer (IARC) conducted a comprehensive review of the association between coffee consumption and cancers of all sites [27]. The IARC Working Group concluded that drinking coffee was “not classifiable as to its carcinogenicity to humans (Group 3).” The Working Group found that coffee was unlikely to cause certain cancers, including cancers of the prostate, pancreas, and breast. Furthermore, coffee consumption might be associated with reduced risk of liver and uterine cancers.

Further evidence since 2016 has confirmed that coffee is not carcinogenic and may indeed reduce the risk of some cancers. A pooled analysis of data from 19 epidemiologic studies, published in 2022, showed that coffee drinkers had a 13% lower risk of being diagnosed with endometrial cancer, and the association was dose-dependent, with more coffee being associated with lower risk of endometrial cancer [9]. Likewise, a meta-analysis of 18 studies found that drinking coffee was associated with a substantially lower risk of hepatocellular carcinoma [28]. An umbrella review of diet and cancer risk at 11 anatomical sites found that coffee consumption was associated with a lower risk of liver cancer and skin basal cell carcinoma [29]. Most recently, the World Cancer Research Fund (WCRF) recommended including “calcium-containing foods (such as dairy products) and coffee in dietary pattern” to prevent colorectal cancers [30].

**Injuries and Accidents**: Large prospective cohort studies have shown that drinking coffee is associated with a lower risk of injuries and accidents [15,31]. This finding is not surprising, given that coffee drinking increases alertness and is associated with higher mobility. Injury and accident risk have been shown to be lower in both drivers and in the risk of falls among the elderly. A case–control study published in BMJ showed that drivers who consumed caffeinated substances to stay awake had a 63% lower risk of crashing (odds ratio = 0.37) than drivers who did not take caffeinated substances [32]. Drinking caffeinated coffee was associated with a 25% to 30% lower risk of fall in older adults in two cohort studies in Spain and in the UK [33].

**Respiratory diseases**: Several large-scale prospective studies have shown inverse associations between coffee drinking and the risk of chronic respiratory diseases or death from respiratory diseases [15,17,21,34]. A meta-analysis of existing studies, published in 2019, found a non-linear inverse association of coffee consumption with mortality from respiratory disease [24]. Since then, several other papers have found inverse associations between coffee consumption and respiratory diseases [31,35,36], bolstering the evidence for consistency of this association.

**Diabetes**: A meta-analysis of 30 prospective studies, published in 2018, included nearly 1.2 million study participants with over 53,000 incident cases of type 2 diabetes [37]. The results showed a substantial reduction (29% reduction, or RR = 0.71) of type 2 diabetes among coffee drinkers. The risk decreased by almost 6% for each additional cup of coffee, and the results were similar for both caffeinated and decaffeinated coffee. Subsequent meta-analyses, published in 2019 [24] and 2021 [38], confirmed these findings.

Furthermore, a meta-analysis of 10 cohort studies showed that diabetics who drink coffee are at lower risks of overall mortality, cardiovascular mortality, and total cardiovascular events [39]. In other words, coffee consumption is associated with a reduced risk of diabetes and is also associated with reduced risk of future death and cardiovascular events among diabetics.

**Liver diseases**: The association of coffee intake with the incidence of liver diseases has been controversial. Earlier studies and a meta-analysis [40] found lower risks of non-alcoholic fatty liver disease (NAFLD) and liver fibrosis in coffee drinkers. However, a more recent meta-analysis and umbrella review did not find a lower risk of NAFLD among coffee drinkers, but it found a substantially reduced risk of fibrosis among NAFLD patients [41]. Randomized trials have not shown an overall reduction in liver enzymes, including Alanine Aminotransferase (ALT), Aspartate Aminotransferase (AST), Gamma-Glutamyl Transferase (GGT), or Alkaline Phosphatase (ALP), but have found an increase in adiponectin [42], a hormone secreted by adipocytes that has anti-inflammatory and anti-fibrotic effects [43].

**Kidney disease**: Studies conducted within the Atherosclerosis Risk in Communities (ARIC) prospective cohort study have shown that coffee consumption is associated with a reduced risk of chronic kidney diseases [44] and acute kidney injury [45]. In these studies, drinking two or more cups of coffee per day was associated with an almost 15% reduction in the risk of chronic and acute kidney problems. Meta-analyses of epidemiologic studies have confirmed that coffee consumption is associated with a reduced risk of chronic kidney disease, end-stage renal disease, and death from kidney diseases [46,47].

**Cognitive disorders**: A recently published meta-analysis of 22 prospective studies and 11 case–control studies showed that both coffee and tea consumption were linked to an approximately 25% lower risk of cognitive disorders. The relationship was non-linear. The lowest risk of Alzheimer’s disease was seen in those who consumed approximately 2.5 cups of coffee per day [48].

**Parkinson’s Disease (PD)**: The associations between intake of coffee and caffeine with the incidence of PD and its progression have been studied in several major prospective studies [49,50,51]. A meta-analysis of these studies found that those who regularly consumed coffee/caffeine had a lower incidence of Parkinson’s disease [52], and patients with PD who regularly consumed coffee/caffeine had a lower rate of disease progression [52]. While multiple mechanisms have been offered for such protective effects of coffee/caffeine [53], the primary mechanism appears to be caffeine’s blockade of A2A receptors [54].

**All outcomes**: An umbrella review of 201 meta-analyses of observational studies with 67 unique outcomes concluded that “coffee consumption was more often associated with benefit than harm for a range of health outcomes across exposures including high versus low, any versus none, and one extra cup a day. … Summary estimates [indicated] largest relative risk reduction at intakes of three to four cups a day versus none” [55]. The findings of this umbrella review are consistent with the overall observation of lower risk of all-cause mortality among coffee drinkers and with the recent FDA ruling.

## 5. Potential Mechanisms Responsible for Improved Health

Multiple mechanisms may explain the observed improved health and increased longevity among coffee drinkers. Some of these mechanisms have been primarily studied in animal models, and others have been studied in large-scale human studies as well. Here, we discuss five salient mechanisms that have been studied in humans: namely, glucose balancing effects, increased physical activity, increased fat oxidation, improved lung function, and reduced inflammation.

**Glucose balancing effects**: Short-term studies have suggested that consumption of coffee, particularly caffeinated coffee, may improve glucose tolerance. The results of a 16-week trial in overweight men with elevated fasting plasma glucose showed that caffeinated coffee consumption decreased the 2-h concentration and the area under the curve of glucose, while such effects were not observed for decaffeinated coffee or no coffee groups [56]. Waist circumference was also reduced in the caffeinated coffee group, but such an effect was not observed in the two other groups [56]. Studies have suggested that the long-term health of liver and beta cell function may be responsible for the lower risk of type 2 diabetes in habitual coffee drinkers [57].

**Increased physical activity**: A randomized, case cross-over trial compared individuals on days that they were randomly assigned to take caffeinated coffee versus days that they were randomly assigned not to take coffee. Caffeinated coffee intake resulted in an average increase of approximately 1000 steps per day [58], which could lead to a substantial reduction in risk of mortality. A study of 16,000 older participants showed a 41% reduction in the mortality rate in those who walked 4400 steps per day compared to those who walked 2700 steps per day [59]. Increased physical activity leads to lower body mass index, hence lower risk of diabetes, and subsequently lower risk of death due to cardiovascular diseases and other causes [60,61]. Similarly, increased physical activity leads to lower risk of frailty, which is very important in avoiding falls and other catastrophic life events, especially in the elderly. In fact, drinking caffeinated coffee has been associated with a lower risk of frailty in older adults [62,63].

**Increased fat oxidation**: Pre-exercise caffeine intake, from coffee or other sources, may lead to increased fat oxidation during exercise and a reduction in body fat composition. The results of two meta-analyses have shown that pre-exercise caffeine intake increased fat utilization and oxygen uptake during aerobic exercise of submaximal intensity, in both fasting [64] and fed [65] states.

**Improved lung function**: Caffeine is structurally related to theophylline, a medication used to treat asthma, acting as a bronchodilator and reducing respiratory muscle fatigue [66]. A Cochrane Database Systematic Review involving seven randomized trials with a total of 75 asthma patients showed a modest improvement in forced vital capacity (FVC), forced expiratory volume in one second (FEV1), and mid-expiratory flow rates for a few hours after coffee consumption [67]. Likewise, a study of over 10,000 people in the ARIC Cohort showed that coffee drinkers had modestly higher FVC and FEV1, but this effect was limited to never-smokers and those who had quit smoking for a long time. It is likely that smoking, which was correlated with coffee drinking, could potentially mask the beneficial effects of coffee drinking on lung function [68]. Two caffeine metabolites, theophylline and paraxanthine, may contribute to the beneficial effects of coffee on the lungs [69].

**Reduced inflammation**: A study of over 1700 adults showed that coffee drinkers had lower levels of immune and inflammatory response. Five markers had lower levels among coffee drinkers: IFNγ, CX3CL1/fractalkine, and CCL4/MIP-1β, which are involved in host response, namely chemotaxis of monocytes/macrophages; sTNFRII, a proinflammatory cytokine; and FGF-2, a regulator of cell growth [70]. Likewise, a study of nearly 1400 individuals showed that coffee drinkers had lower plasma levels of C-reactive protein (CRP) and E-selection, an inducible endothelial cell surface molecule that has an important role in inflammatory reaction and development of vulnerable plaques [71]. Studies have suggested that lower subclinical inflammation, as indicated by lower levels of CRP, may partially mediate the association between coffee consumption and lower risk of diabetes [72,73].

## 6. The Role of Coffee Types and Coffee Additives

Where data was available, studies have assessed the association of caffeinated and decaffeinated coffee consumption with overall mortality. In almost all such analyses, both caffeinated and decaffeinated coffee were associated with reduced risk of mortality [15,17,18,20,21,22]. However, individuals who drank decaffeinated in the year prior to the study might have been caffeinated coffee consumers previously, leading to a potential misclassification of the beneficial effects of caffeinated coffee for decaffeinated coffee.

At least one study has examined the association of ground coffee with mortality [22]. This study found that all types of coffee, including ground coffee, were associated with reduced mortality.

Adding sugar and cream may modify the effect of coffee drinking on health. For example, Zhang et al. found that unsweetened coffee substantially reduced the risk of neurodegenerative diseases and their related mortality [49], but sweetened coffee was not associated with the risk of neurodegenerative diseases. In other words, adding sugar may nullify the beneficial effects of coffee. In yet another study, Narita and colleagues found that black coffee was associated with a reduced risk of depression, while sugar-sweetened coffee had the opposite effect [74]. A study combining three Harvard prospective cohort studies showed that increased consumption of unsweetened caffeinated and decaffeinated coffee was inversely associated with weight gain, but the addition of sugar to coffee counteracted coffee’s benefit for possible weight management [75]. In this study, adding cream or coffee whitener was not associated with greater weight gain. Likewise, a recent study of over 46,000 adults enrolled in NHANES showed that mortality benefits were restricted to black coffee and coffee with low added sugar and saturated fat, but higher amounts of these additives nullified the beneficial effects [76]. However, other studies have found that even sweetened coffee is associated with health benefits. For example, Loftfield and colleagues [17] found that hazard ratio estimates did not differ between persons who drank their coffee black and those who used common coffee additives, such as milk, cream, and sugar.

Currently, the FDA allows coffee to be labeled as ‘healthy’ only if it contains fewer than 5 calories per Reference Amount Customarily Consumed (RACC) [10].

## 7. Areas of Potential Concern

There is now compelling evidence from human studies that coffee drinkers have lower risk of most major causes of mortality. However, there have traditionally been some concerns about health risks associated with coffee drinking. The primary areas of concern have been increased risk of cancer, hypertension, cardiac arrhythmias, pregnancy outcomes, and mental health. Some of these concerns are now resolved, but there are still some lingering issues with other ones.

**Cancer**: As discussed earlier, current data from very large epidemiologic studies show that coffee drinkers are not at an increased risk of cancer. Coffee may indeed be associated with reduced risk of several cancers, such as cancers of the liver, uterus, and skin [9,28,29]. However, there have been lingering concerns about the potential carcinogenicity of coffee, partly because of studies conducted nearly four decades ago that turned out to be methodologically incorrect, and partly because of some constituents of coffee. A well-known study, published in 1981, showed a strong association between coffee drinking and cancer of the pancreas [77]. The results of this study were widely disseminated, even making headlines in the New York Times [78]. However, it was later found that the study selected an inappropriate control group, which highly biased the results. This study did, in fact, become a textbook example of how an epidemiologic study could go wrong. Subsequent large-scale prospective studies convincingly showed that coffee drinking was not associated with any increased risk of pancreatic cancer [79] or other cancers [18].

**Hypertension**: Drinking coffee results in short-term increases in blood pressure. However, the results of epidemiologic studies do not support any long-term increase in the risk of hypertension in coffee drinkers. In fact, the results of recent meta-analyses showed a 7% reduction in the risk of hypertension when the results of 13 cohort studies were combined [80]. This reduction was more substantial when the results of cross-sectional studies were combined [80]. A recent position paper by the International Society of Hypertension, endorsed by the World Hypertension League and European Society of Hypertension, concluded that “moderate regular coffee consumption (three to four cups per day) does not adversely affect blood pressure and the cardiovascular system and can be moderately beneficial” [81].

**Arrhythmias**: Klatsky and colleagues studied 130,054 persons who received care from Kaiser Permanente in California, of whom 3137 were hospitalized for cardiac arrhythmias [82]. Using Cox regression models adjusted for eight covariates, they found that coffee drinkers had a lower risk of hospitalization due to arrhythmias, with HRs of 0.9 for 1–3 cups/day and 0.8 for ≥4 cups/day (*p* for trend = 0.002). These investigators concluded that “the inverse relations of coffee and caffeine intake to hospitalization for arrhythmias make it unlikely that moderate caffeine intake increases arrhythmia risk”. Other studies have generally reached similar conclusions. A double-blind, randomized clinical trial with a cross-over design conducted at a tertiary-care university hospital found that “acute ingestion of high doses of caffeine did not induce arrhythmias in patients with systolic heart failure and at high risk for ventricular arrhythmias” [83].

**Pregnancy outcomes**: Coffee is a major source of caffeine, which at high doses may negatively affect pregnancy outcomes. However, lower caffeine doses during pregnancy may be safe. Leading health organizations, such as the American College of Obstetricians and Gynecologists (ACOG) and the European Food Safety Agency (EFSA), report that caffeine consumption in doses up to 200 mg/day does not raise safety concerns in pregnant women [84,85].

Physiological concerns about caffeine consumption stem from the possibility of increased catecholamine concentrations, which may lead to vasoconstriction, less fetal growth, and increased cAMP, affecting cellular development. In parallel, pregnancy reduces the activity of hepatic cytochrome P450 1A2 (CYP1A2), resulting in a nearly three-fold increase in the half-life of caffeine among pregnant women [86].

Meta-analyses of observational studies have found approximately a 38% increased risk of low birth weight in women who drink coffee during pregnancy [87]. However, this association is significantly affected by several confounding variables. For example, increased coffee consumption is also associated with increased alcohol consumption, smoking, and lower education levels [15], which are in turn associated with undesirable pregnancy outcomes. Another key confounding variable is the “pregnancy signal”, wherein women with weaker placentas have both less aversion to coffee consumption and higher rates of pregnancy complications [88]. Additionally, recall bias—the tendency for women with pregnancy complications to remember what they may perceive as problematic behavior—may inflate such associations [89].

Some randomized trials have found no association between coffee/caffeine consumption and poor pregnancy outcomes. Notably, in a randomized controlled trial in Denmark (*n* = 1207), pregnant mothers were assigned to consuming caffeinated or decaffeinated coffee. The caffeinated group, on average, consumed 182 mg more caffeine per day. This study found no significant differences between the two groups in terms of birthweight, gestational weeks of pregnancy, birth height, or other outcomes [90].

In sum, while large doses of caffeine may be potentially problematic during pregnancy, there is little evidence suggesting that caffeine doses under 200 mg/day have any significant effect on pregnancy outcomes.

**Mental Health**: Many papers have been published on the association of coffee/caffeine intake with depression, anxiety, and other mental health outcomes. In general, meta-analyses suggest that coffee is associated with a reduced risk of depressive symptoms [91,92,93]. Coffee may reduce depressive symptoms via its antioxidants (such as polyphenols), its caffeine content, reducing inflammation, or changing gut microbiota. Studies have shown that those who regularly consume polyphenol-rich drinks, such as coffee or tea, are less likely to have high levels of depressive symptoms or perceived stress [94]. However, when taken at excessive doses, coffee may be anxiogenic and cause panic attacks, particularly in patients with panic disorder [95]. In fact, symptoms caused by coffee/caffeine intake, when taken at excessive doses—such as tachycardia, palpitation, restlessness, and tremor—are not distinguishable from anxiety attacks. “Excessive use” is dependent on each person. Therefore, each individual needs to personalize coffee/caffeine intake.

## 8. Well-Being Outcomes of Interest

Coffee is often taken to hydrate the body, enhance sports performance, improve mental acuity, and delay or reduce sleep. It may also increase bowel movement.

**Hydration**: Despite the diuretic effect of its caffeine content, coffee is a useful source of hydration, likely comparable to water. Caffeine intake, in excess of 250 mg, leads to a slightly increased urine output. This effect is limited to consuming large doses, populations with lower caffeine tolerances, and only when caffeine ingestion is not followed by exercise [96,97]. In a counterbalanced cross-over trial of 50 coffee drinkers, Killer and colleagues found no significant difference in total body water, 24-h urine volume, or key hematological markers between drinking 800 mL of coffee and a similar volume of water [98]. Furthermore, Maughan and colleagues have shown that the total urine mass after 4 h of drinking coffee is comparable to water [99]. Likewise, the beverage hydration index of coffee is not significantly different from the still-water control [99].

**Sports Performance**: Ergogenic effects of coffee/caffeine intake have been studied for over 100 years [100]. The International Society of Sports Nutrition reports that studies show a small to moderate enhancement in exercise performance, with this effect varying among individuals [101].

**Mental Acuity**: When consumed at low to moderate doses, caffeine blocks adenosine receptors, leading to enhanced alertness, vigilance, and attention, as well as shortened reaction time [102]. Coffee/caffeine consumption may also enhance individual senses, such as visual acuity [103]. For example, a placebo-controlled, double-blind, balanced cross-over study showed that low-dose caffeine consumption improved accuracy for both the horizontal and random motion paths of dynamic visual acuity [103].

**Sleep**: It is well known that coffee increases alertness and delays sleepiness. A well-conducted randomized, case cross-over trial [58] showed that coffee intake may lead to approximately 36 min of less sleep each day. Similarly, a systematic review and meta-analysis of the literature showed that coffee consumption reduced total sleep time by 45 min and sleep efficiency by 7% [104]. This study suggested that “to avoid reductions in total sleep time, coffee (107 mg per 250 mL) should be consumed at least 8.8 h prior to bedtime and a standard serve of pre-workout supplement (217.5 mg) should be consumed at least 13.2 h prior to bedtime” [104].

**Bowel movement**: Lower duration of postoperative ileus after colorectal surgery improves patient recovery and reduces hospital costs. Several studies, both randomized and non-randomized, have evaluated the effect of coffee consumption on postoperative ileus. The results of a recent meta-analysis, with a total of 610 patients, showed that coffee/caffeine intake significantly reduced time to first bowel movement and time to first solid food intake after elective laparoscopic colorectal surgery, while time to first flatus and length of hospital stay did not change significantly [105].

## 9. Research Trends and Future Directions

The initial epidemiologic studies investigating the impact of coffee on health had primarily retrospective case–control designs. Over the past two decades, large-scale prospective cohort studies have provided substantial information on the effects of coffee on health. Some notable examples in the United States and Europe include, but are not limited to, the Nurses’ Health Study, Nurses’ Health Study II, Health Professionals Follow-Up Study, the NIH-AARP Study, the UK Biobank Study, and the European Prospective Investigation into Cancer and Nutrition (EPIC) [15,18,20,22]. Likewise, substantial information about coffee and health has been provided through prospective cohort studies conducted in Japan, China, and other Asian countries [23]. The results have been consistent. Several million individuals have been followed in these studies for over two decades. Therefore, this field is relatively saturated, and it is unlikely that prospective cohort studies add substantial new information. If any new cohorts are developed, or if repeated data collection is considered, it may be useful to ask detailed questions about types of coffee drinking (e.g., caffeinated vs. decaffeinated), added cream and sugar, consumption of ground coffee vs. other types, etc. These questions should try to minimize contamination by asking about consumption over the past few decades, rather than just the year prior.

Over the past decade, a large number of Mendelian Randomization (MR) studies have been published to assess the effects of coffee on health. Many of these studies used data from the UK Biobank Study. MR studies have significant strengths, including lower confounding, avoidance of reverse causation, cost-effectiveness by leveraging existing genetic data, and serving as a complement to observational studies. Nevertheless, MR studies have their own weaknesses, including three key assumptions that may not be met, i.e., (1) the genetic variant is robustly associated with the exposure; (2) the genetic variant influences the outcome only through the exposure (exclusion restriction); and (3) the genetic variant is not associated with confounders [106,107]. For example, the genetic variants may be only weakly associated with the exposure, which biases the results toward null. Therefore, MR studies may not resolve controversies. However, it is reassuring that the results of MR studies have generally been consistent with the results of large-scale observational studies. For example, in the UK Biobank Study, there was no overall association between genetically predicted coffee consumption with the overall risk of being diagnosed with or dying from cancer [108], a finding that is consistent with observational studies.

It is important to conduct large-scale randomized studies of coffee/caffeine consumption, with collection of detailed data, biological samples, and other biometric measures. There have been numerous randomized studies with parallel or cross-over designs, but many of these studies had small sample sizes, were short term, and focused on a very narrow scope of outcomes. The fact that one of these studies, quite well-designed, was recently published in the New England Journal of Medicine [58], is a testament to the need for such studies. Perhaps larger-scale studies, with longer-term follow-up times, measuring an array of outcomes, will be very helpful.

## 10. Summary and Conclusions

Coffee, once regarded with skepticism, has emerged as a beverage with a broad range of potential health benefits. Mounting evidence from large-scale epidemiological studies consistently shows that moderate coffee consumption is associated with reduced all-cause and cause-specific mortality, as well as with lower risks of major chronic diseases, including cardiovascular diseases, type 2 diabetes, certain cancers, cognitive decline, and respiratory illnesses. These benefits often persist across diverse populations.

Mechanistically, coffee’s protective effects are likely mediated through its impact on improved glucose balance, enhanced physical activity, higher fat oxidation, anti-inflammatory properties, and enhancement of metabolic and pulmonary functions. While concerns persist regarding pregnancy outcomes, anxiety, and sleep disturbances—particularly with high consumption levels—most data support that moderate coffee intake (3–5 cups/day) is safe for the general population and beneficial for most.

Coffee also improves some other aspects of well-being, such as mental alertness, physical performance, and hydration, further enhancing its role in daily functioning and well-being. Although most evidence comes from observational studies, the consistency of findings across settings adds to their credibility. Future research—especially randomized controlled trials and genetic studies—will help clarify unanswered questions and identify subgroups that may benefit most or require caution. In sum, coffee, when consumed in moderation and tailored to individual tolerance, appears to be a health-promoting beverage.

## Figures and Tables

**Table 1:** Coffee consumption and overall mortality in large-scale prospective cohort studies.

Authors (Year)	Cohort Study	Number of Participants (Rounded to 10,000)	Geographic Area	Consumption Associated with the Lowest Risk	Multivariable Adjusted Hazard Ratios (HRs)
Klatsky et al. (1993) [11]	KPMHC	130,000	United States	>6 cups/day	0.88
Kleemola et al. (2000) [12]	---------	20,000	Finland	4–7 cups/day	0.61 in men 0.49 in women
Andersen et al. (2006) [13]	IWHS	30,000	United States	4–5 cups/day	0.81
Tamakoshi et al. (2011) [14]	JACC	100,000	Japan	2–3 cups/day	0.86 in men 0.83 in women
Freedman et al. (2012) [15]	NIH-AARP	400,000	United States	4–5 cups/day	0.88 in men 0.84 in women
Liu et al. (2013) [16]	ACLS	40,000	United States	2–3 cups/day	1.04 in men 0.85 in women
Loftfield et al. (2015) [17]	PLCO	90,000	United States	4–5 cups/day	0.79
Ding et al. (2015) [18]	NHS, NHS II, and HPFS	200,000	United States	3–5 cups/day	0.94 in all 0.84 in never-smokers
Löf et al. (2015) [19]	---------	50,000	Sweden	2–5 cups/day	0.81
Gunter et al. (2017) [20]	EPIC	520,000	Europe (10 countries)	≥3 cups/day	0.82 in men 0.92 in women
Park et al. (2017) [21]	MEC	180,000	United States	≥2 cups/day	0.82
Loftfield et al. (2018) [22]	UK Biobank	500,000	United Kingdom	6–7 cups/day	0.84
Shin et al. (2022) [23]	ACC	530,000	Asia (4 countries)	3–5 cups/day	0.76 in men 0.65 in women

HRs are in comparison to no coffee consumption or the lowest level of consumption. KPMHC: Kaiser Permanente Multiphasic Health Checkup Cohort Study; IWHS: Iowa Women’s Health Study; JACC: Japan Collaborative Cohort Study for Evaluation of Cancer Risk; NIH-AARP: The National Institutes of Health-AARP Diet and Health Study; ACLS: Aerobics Center Longitudinal Study; PLCO: The Prostate, Lung, Colorectal, and Ovarian Cancer Screening Trial; NHS: Nurses’ Health Study; HPFS: Health Professional Follow-Up Study; EPIC: European Prospective Investigation into Cancer and Nutrition; MEC: Multi-Ethnic Cohort; ACC: Asian Cohort Consortium, comprising 12 cohort studies conducted in Asia.
